# Machine Learning in Neurosurgery: Toward Complex Inputs, Actionable Predictions, and Generalizable Translations

**DOI:** 10.7759/cureus.51963

**Published:** 2024-01-09

**Authors:** Ethan Schonfeld, Nicole Mordekai, Alex Berg, Thomas Johnstone, Aaryan Shah, Vaibhavi Shah, Ghani Haider, Neelan J Marianayagam, Anand Veeravagu

**Affiliations:** 1 Neurosurgery, Stanford University School of Medicine, Stanford, USA; 2 Medicine, Tel Aviv University, Tel Aviv, ISR; 3 School of Humanities and Sciences, Stanford University, Stanford, USA

**Keywords:** ai and robotics in healthcare, ai and machine learning, outcome prediction, generative adversarial network (gan), generative ai, federated learning, spine, neurosurgery

## Abstract

Machine learning can predict neurosurgical diagnosis and outcomes, power imaging analysis, and perform robotic navigation and tumor labeling. State-of-the-art models can reconstruct and generate images, predict surgical events from video, and assist in intraoperative decision-making. In this review, we will detail the neurosurgical applications of machine learning, ranging from simple to advanced models, and their potential to transform patient care. As machine learning techniques, outputs, and methods become increasingly complex, their performance is often more impactful yet increasingly difficult to evaluate. We aim to introduce these advancements to the neurosurgical audience while suggesting major potential roadblocks to their safe and effective translation. Unlike the previous generation of machine learning in neurosurgery, the safe translation of recent advancements will be contingent on neurosurgeons’ involvement in model development and validation.

## Introduction and background

Artificial intelligence (AI) utilizes computer systems to simulate human cognitive abilities. Machine learning (ML), a domain of AI, enables algorithms to recognize patterns in large, complex datasets to produce predictive outputs at inference time. Biomedical data has become vast and complex, thereby requiring AI to identify clinically meaningful relationships [1]. Hospitals produce large quantities of unstructured data from monitoring devices, imaging, and patient notes, requiring novel methods to help physicians effectively analyze this "big data" to identify predictive relationships. Such novel ML methods applied to certain tasks have outperformed not only classical statistical models but also clinical experts [1].

ML techniques have aided all neurosurgical domains, including early diagnosis, clinical decision-making, patient management, drug discovery, and prognosis prediction. In the case of outcome prediction, there has been an average accuracy of 0.945 and an average area under the curve (AUC) of 0.83, spanning across predictions for functional, spine, neurovascular, brain tumor, and traumatic subdomains [1]. This average performance of ML models was found to be significantly better than logistic regression models (median absolute performance increase of 15% accuracy) [1]. While simple ML models like logistic regression dominate most published applications to neurosurgery, this performance achievement of ML resulted from more complex models [1]. Such complex models (e.g., deep neural networks (DNNs), convolutional neural networks (CNNs)) have allowed for the utilization of complex input types including imaging and real-time surgical video, and thereby the prediction of complex outputs including non-radiographic intraoperative measurements of Cobb angle [2], cerebral artery segmentation in operative field of view [3], and augmented reality guidance for catheter placement for external ventricular drains [4].

With the attention toward AI/ML in neurosurgery significantly increasing, the National Institutes of Health funded 535 projects applying ML to clinical research, representing $264 million in 2017 [5]. Attitudes in neurosurgery were open to the application of AI in neurosurgery for imaging interpretation, operative planning, autonomous surgery, and hazard/complication prediction [6]. Despite funding, open attitudes, and substantial research efforts, few technological applications have been integrated into patient care. While many algorithms have achieved high predictive power for outcome predictions, few have been externally validated across multiple sites. This is worrisome because unlike fields of autonomous driving and search engine development in which data diversity is high, neurosurgical data used to train ML algorithms is generally from single institutions.

In this comprehensive narrative review, our objective is to acquaint clinical practitioners, who will play a pivotal role in the safe and efficient integration of this technology, with the nuances of ML in the context of neurosurgery. We explore the diverse algorithms and their applications in this field, while also addressing the technical challenges that may impede their effective implementation in enhancing patient care. A particular emphasis is placed on the critical aspects of data sharing and the necessity of training models using diverse data sets. We discuss these elements in depth, offering both technical insights and clinical perspectives, to underscore their importance in ensuring the safe and successful adoption of ML in neurosurgery.

Definitions of technologies

The AI technologies in this review are described in Table 1.

**Table 1:** Technology definitions AI: Artificial intelligence; ML: Machine learning; ANN: Artificial neural networks

Technology	Definition
Artificial intelligence (AI)	Simulation of problem-solving and logical thinking by computer systems to perform tasks.
Machine learning (ML)	Subfield of AI that learns patterns in large data without explicit instruction that are often used to make predictions.
Deep learning (DL)	Subfield of ML that uses ANNs to mimic the learning process of the human brain.
Artificial neural networks (ANN)	Systems of artificial neurons trained to learn complex interactions within input data to optimize a cost/objective function.
Supervised learning	Subfield of ML that trains models using labeled datasets to predict labels for similar data.
Unsupervised learning	Subfield of ML that learns patterns in unlabeled data that can be used to label, classify, and represent the data.

## Review

Input variable types and data sources

With many ML model types and potential inputs, careful attention to input data type should help determine model selection during development. Table 2 covers input data types that are compatible with the different ML models. For the training of such models, Table 3 details publicly available datasets, focusing on those most frequently used and those serving as examples of emerging dataset releases with large potential impacts.

**Table 2:** Machine learning method types and input variables

	Input variables	Example use case
Logistic regression	Features (i.e., clinical features)	Prediction of outcomes following aneurysmal subarachnoid hemorrhage using clinical information, neuroimaging features (i.e., clot size), and treatment modality information (Feghali et al., 2022)[7]
Support vector machine (SVM)	Features (i.e., clinical features)	Differentiation of benign and malignant pediatric brain tumors using blood markers (Khayat Kashani et al., 2022)[8]
Artificial neural networks (ANN)	Features (i.e., clinical features)	Prediction of traumatic brain injury outcomes in low-resource settings from easily acquired clinical variables (Adil et al., 2022)[9]
Convolutional neural network (CNN)	Imaging, video, genomics, time series	Intraoperative microscopic tumor detection from label-free stimulated Raman scattering microscopy (Reinecke et al., 2022)[10]
Long short–term memory network (LSTM)	Free text, genomics, time series	Differentiating lumbar disc herniation and lumbar spinal stenosis using free text admission notes (Ren et al., 2022)[11]
Transformer	Free text, imaging, genomics, time series	Classification and histopathological feature labeling of primary brain tumors (Li et al., 2023)[12]
Generative adversarial network (GAN)	Imaging	Generating CT from MRI data (Ranjan et al., 2021)[13]
Foundational model	Broad data	Adding realistic–looking abnormalities to synthetically generated radiology images (Chambon et al., 2022)[14]

**Table 3:** Selected publicly available databases and datasets used to develop machine learning models for neurosurgery and medical applications MIMIC-III: Medical Information Mart for Intensive Care-III; VerSe: Large Scale Vertebrae Segmentation Challenge; SRH: Stimulated Raman histology; ICD: International Classification of Diseases; CM: Clinical modification; CPT: Current Procedural Terminology; eICU: Electronic intensive care unit

Dataset	Data collection period	Included labels	Number of subjects
Truven Health Analytics MarketScan research database[15]	2010-2018	Demographic, admission, diagnosis codes, discharge status, procedure codes, length of stay, financial information, drug information	~273 million
National (Nationwide) Inpatient Sample (NIS) discharge database[16]	2012-2022	Demographic, admission, diagnosis codes, procedure codes, financial information, disease severity, hospital information, length of stay	~35 million
MIMIC-III[17]	2008-2014	Demographics, clinical measurement, intervention, billing, pharmacotherapy, clinical laboratory test, medical data	~38,000 distinct adult patients ~53,000 admissions
VerSe[18,19]	2013-2017	CT imaging, annotated vertebrae (segmentation mask, fracture grading)	374 CT scans, 355 patients
OpenSRH[20]		Clinical stimulated Raman histology images, full pathologic annotations, whole slide tumor segmentations, raw and processed optical imaging data	~300 patients ~1300 whole slide optical images
Parkinson's Progression Marker Initiative (PPMI)[21]	2011-ongoing	Demographics, admission, diagnosis codes clinical, imaging, omics, genetic, sensor, and biomarker data	~ 1758 patients
American College of Surgeons National Surgical Quality Improvement Program (NSQIP-P)[22]	2005-ongoing	Demographic variables, preoperative risk factors, intraoperative variables, and 30-day postoperative mortality	~10 million
The Quality Outcomes Database (QOD)[23]	2012-2019	Demographic, admission, diagnosis codes, discharge status, variables specific to lumbar and cervical pathologies	~120,000
eICU Collaborative Research Database[24]	2014-2015	Demographics, vital sign measurements, care plan documentation, severity of illness measures, diagnosis information, treatment information, admission codes	~200,000
The Surveillance, Epidemiology, and End Results Program (SEER)[25]	2002-Ongoing	Patient demographics, primary tumor site, tumor morphology and stage at diagnosis, first course of treatment, and follow-up for vital status	~10 million
University of Pennsylvania Glioblastoma Imaging, Genomics, and Radiomics (UPenn-GBM)[26]	2006-2018	Clinical, demographic, and molecular information MRI, perfusion, and diffusion derivative volumes, computationally derived and manually revised expert annotations of tumor sub-regions, as well as quantitative imaging features corresponding to each of these region	630 patients
Clinformatics™ DataMart[27]	2007-2019	Clinical and demographic information, Date of service, place of service, ICD-9-CM codes, CPT codes, provider type, drug quantity dispensed, days supplied, charges, deductibles, and copayments	~100 million

Outcome prediction

Postoperative Complications

ML modeling has been applicable in predicting neurosurgical postoperative complications. van Niftrik et al. utilized gradient-boosting ML modeling to predict patients at risk for early complications (AUC = 0.73) following intracranial tumor surgery [28]. Neural networks identified patients with a high risk for cerebrospinal fluid (CSF) leaks following pituitary surgery using clinical and surgical features as input variables, achieving high discrimination (AUC = 0.84) [29]. In spine surgery, 90-day complication following anterior cervical discectomy and fusion has been predicted by DNN (AUC = 0.832) [30], and for both posterior lumbar spinal fusion and adult spinal deformity surgery, specific complications were predicted by artificial neural network (ANN) [31,32].

Quality of Life

ML modeling has been used to predict quality of life based on assessment scores, including the Oswestry Disability Index (ODI) and the Glasgow Outcome Score (GOS). Using ANNs, predictions of GOS, which assess the long-term necessity for rehabilitation, in patients with aneurysmal subarachnoid hemorrhage achieved AUC of 0.85 and 0.96 based on clinical and angiogram data respectively [33]. Azimi et al. used ANNs to predict postsurgical satisfaction over two years for patients with lumbar spinal canal stenosis undergoing lumbar spine surgery [34]. Support vector machine (SVM) predicted postoperative ODI prior to operation for cervical spondylotic myelopathy using preoperative ODI and symptom duration with a coefficient of determination of 0.932 [35].

Recurrence and Mortality

Tumor recurrence has been predicted effectively by ML models. For example, early progression and recurrence outcomes for patients with parasagittal and parafalcine meningiomas were successfully predicted by SVM and random forest (RF), achieving AUC = 0.91 [36]. SVMs were used to predict survival for glioma, achieving similar or higher AUC in comparison to a team of neuroradiologists [37].

Translation of ML in Neurosurgery

A comprehensive analysis of translation efforts in ML applied to neurosurgery is detailed in Table 4.

**Table 4:** Analysis of translation efforts AUC: Area under the curve; CTA: Computed tomography angiography; ICP: Intracranial pressure; MAP: Mean arterial pressure; CSF: Cerebrospinal fluid; LVO: Large vessel occlusion; IB: Imaging biometrics; GBM: Glioblastoma; CT: Computerized tomography; MRI: Magnetic resonance imaging; BMI: Body mass index

Study/device	Externally validated	Applied for FDA approval	Translated into clinic	Type of model	Training method	Input	Outcome	Model performance
Valliani et al.[38]	Yes	No	No	Ensemble learning algorithm	Single center	Clinical features and surgical variables	Non-home discharge after thoracolumbar spine surgery	AUC: 0.81 (test) AUC: 0.77 (external)
Senders et al.[39]	Yes	No	No	Accelerated failure time model	Retrospective dataset	Demographic, socioeconomic, clinical, and radiographic features	Prediction of survival in glioblastoma patients	C-index: 0.70 (best time-to-event model), 0.70 (best continuous and binary model)
Liu et al.[40]	Yes	No	No	Decision tree	Retrospective	Demographic, clinical, and aneurysm-specific features as well as Glasgow Coma Score	Long-term outcomes after poor-grade aneurysmal subarachnoid hemorrhage	AUC: 0.88 (test) AUC: 0.94 (external)
Staartjes et al.[41]	Yes	No	No	Logistic generalized additive model	Multicenter retrospective database	Clinical features and labs	Functional impairment after intracranial tumor surgery	AUC: 0.72 (test), AUC: 0.72 (external)
Viz Aneurysm[42]	Yes	Yes	Yes	Neural network	Unknown	CTA imaging	Detection and analysis of cerebral aneurysms	93.8% sensitivity 94.2% specificity (external)
Carra et al.[43]	Yes	No	No	Random forest	Prospective and retrospective databases	ICP and MAP signals	Doses of harmful intracranial pressure in patients with severe traumatic brain injury	AUC: 0.94 (external)
Aidoc BriefCase-CSF triage[44]	Yes	Yes	Yes	Neural network	Unknown	CTA images	Detection of cervical spine fractures	(external) Sensitivity - 54.9 Specificity - 94.1
Rapid LVO 1.0[45]	Yes	Yes	Yes	Density threshold-based traditional machine learning	Unknown	CTA images	Detection of anterior circulation LVOs	Sensitivity - 96.0 Specificity - 98.0
Imaging biometrics (IB) Neuro-Oncology[46]	Yes	Yes	Yes	Artificial Intelligence	Unknown	CTA images	Distinguishing glioblastoma progression	Classification of survival in GBM sensitivity - 80.0 Specificity - 63.0
Staartjes et al.[47]	Yes	No	No	Logistic regression	Retrospective multicenter database	Clinical features and surgical variables	Prediction of mid-term outcomes after lumbar spinal fusion	Functional Impairment AUC:0.75 (test) AUC:0.67 (external) Back pain AUC: 0.71 (test) AUC:0.72 (external) Leg Pain AUC: 0.72 (test) AUC: 0.64 (external)
Thanellas et al.[48]	Yes	No	No	Convolutional neural network	Retrospective single-center dataset	CTA images	Identification and localization of subarachnoid hemorrhage on CT scans	Development: Sensitivity 87.4% and specificity 95.3% External: sensitivity 99.3% and specificity 63.2%
Teng et al.[49]	Yes	No	No	Logistic regression	Retrospective multicenter database	Clinical features lab values and imaging	Predicting high-grade intracranial meningioma	Development: AUC: 0.99 AUC: 0.75 (internal) AUC: 0.842 (external)
Vitrea CT Brain Perfusion[50]	Yes	Yes	Yes	Convolutional neural network	Unknown	CTA images	Detection of intracranial aneurysms	Sensitivity - 90.9-96.3 Specificity - 100.0
Ma et al.[51]	Yes	No	No	Neural network	Retrospective multicenter database	MRI images	Prediction of intramedullary glioma grade	AUC: 0.8431
Biswas et al.[52]	Yes	No	No	Neural network	Retrospective single-center database	Predictor variables (demographic and lab)	Predicting chronic subdural hematoma referral outcome	AUC: 0.951 (test) AUC: 0.896 (external)
Fang et al.[53]	Yes	No	No	Convolutional neural network	Retrospective single-center database	Demographic, clinical, and imaging variables	Glasgow Outcome Scale	AUC (test): 0.93 AUC (external): 93.69
Karhade et al.[54]	Yes	No	No	Logistic regression	Retrospective multicenter database	Clinical labs and tumor-specific variables	Six-week mortality in spinal metastasis cases	AUC (test): 0.84 AUC (external): 0.81
Crabb et al.[55]	Yes	No	No	Logistic regression, random forest, support vector machine, and ensemble learning algorithm	Public database	Age, BMI, clinical variables, and preoperative lab tests	Prediction of unplanned 30-day readmissions after pituitary adenoma resection	AUC (external): 0.76
Warman et al.[56]	Yes	No	No	Ensemble learning algorithm	Prospective single center dataset and public dataset	Patient demographics, presenting vital signs, mechanism of injury, initial Glasgow Coma Scale (GCS)	Predicting in-hospital mortality after traumatic brain injury	AUC (test): 0.91 AUC (external): 0.89
Habets et al.[57]	Yes	No	No	Logistic regression	Retrospective single center	Patient demographic data, disease-specific data, clinical performance scores, and neuropsychological scores	Prediction of motor response after deep brain stimulation	AUC (test): 0.79 AUC (external) 0.79

Intraoperative and robotic applications

ML approaches underpin many of the robotic functions that currently drive improvements in patient outcomes in neurosurgery. With these techniques, neurosurgeons retain accuracy over the course of long, technically complex operations, where minuscule reductions in operative times, patient length of stay, revision surgeries, and morbidity translate to large amounts of value added [58]. Spinal robots have improved pedicle screw placement accuracy (odds ratio (OR) 0.44 compared to freehand; OR 0.50 compared to CT navigation) and optimal screw placement [59,60], while intracranial automation has increased the speed and accuracy that leads to deep brain stimulation and stereoelectroencephalography are placed [61]. The exciting capabilities of neurosurgical robotics augmented with ML are image registration for intraoperative neuronavigation and surgical task automation, enhancing operative planning, efficiency, and accuracy.

Neuronavigation

Neuronavigation, the application of technology to localize lesions within the skull or vertebral column, has drastically impacted workflow within various neurosurgical subdisciplines, driving improvements in outcomes from epilepsy surgery to spinal fusion. AI systems have served important roles in traditional neuro-navigation systems, using infrared, electromagnetism, and ultrasonography methods to track position. Currently, novel ML methods are being developed to enhance these traditional systems. A key challenge in intraoperative neuronavigation is high-fidelity image registration, or the action of updating preoperative imaging to reflect real-time changes in the surgical field, while creating navigation plans. For example, resection of a large tumor can significantly alter the patient’s anatomy, such that the preoperative imaging used to create the navigation plan is no longer accurate [62]. Intra-operative registration updates the navigation plan to reflect the amount of tumor removed and the shift of normal tissue toward the resection cavity. Several models have been proposed to optimize registration; Han et. al. have developed an unsupervised, dual-channel deep learning (DL) network that updates preoperative magnetic resonance imaging (MRI) with intraoperative computerized tomography (CT) [63]. Such registration techniques can improve current robot navigation systems, enhancing the speed and accuracy of neurosurgical operations.

Subtask Automation

While neurosurgical procedures require a skilled surgeon, some tasks may be automated, enabling attention to critical areas. During an awake craniotomy, a neurosurgical robot could automatically stimulate a critical area of the eloquent cortex, sensing when the operator has performed the delicate part of the operation. Padoy and Hager have demonstrated such surgeon-robot interactions with Hidden Markov models; they enabled a da Vinci surgical system (Intuitive Surgical, Sunnyvale, United States) to recognize when a surgeon had inserted a suture needle, automatically pull the needle through, and seamlessly transfer control back to the surgeon for the next bite [64]. Similarly, Hu et al. indicated that the RAVEN II Surgical Robot could semi-autonomously perform brain tumor ablation via modeling and implementation of a behavior tree framework [65]. Robotics augmented with ML algorithms can further automate surgical trajectory planning as demonstrated by the minimally invasive RAVEN II Surgical Robot that can plan efficient trajectories to approach and resect residual brain tumor at its margins following debulking [66]. Regarding a stereotactic brain biopsy, neurosurgeons using computer-assisted route planning software were able to create shorter trajectories that were more orthogonal to the skull and carried a lower risk of injuring vasculature as compared to manually planned routes [67].

Non-robotic Intra-operative Applications

ML has non-robotic intraoperative uses that may improve neurosurgical practice. Wong et al. developed an unsupervised ML model that used microelectrode recording inputs to functionally localize and visualize the subthalamic nucleus (STN) during deep brain stimulation (DBS) procedures [68]. Valsky et. al reported their successfully trained Hidden Markov model that identifies when a DBS lead improperly exited the ventral STN [69]. It is also possible to predict postoperative motor improvement in Parkinson’s disease following DBS by applying an RF to intraoperative microelectrode recording data [70]. Beyond functional neurosurgery, Jermyn et. al showed that an RF classifier could be applied to intraoperative Raman spectroscopy data during grade 2-4 glioma resection, identifying more invasive cells with an accuracy of 92% while a surgeon with a bright-field microscope and MRI could identify the same cancer cells with 73% accuracy [71]. More recently, intraoperative high-resolution magic angle spinning nuclear magnetic resonance was used in conjunction with an RF to differentiate tumor cells from healthy controls with a median AUC of 85.6% and area under the precision-recall curve (AUPR) of 93.4, while also being able to distinguish between benign and malignant samples with a median AUC of 87.1% and AUPR of 96.1% [72]. Ritschel et al. showed that SVMs trained on contrast-enhanced ultrasound image data can accurately detect the resection margins of glioblastoma [73]. Li et al. developed an approach where a CNN with a long short-term memory architecture was applied to probe-based confocal laser endomicroscopy images to successfully differentiate between glioblastoma and meningioma [74].

Overall, clinically useful applications of ML to neurosurgical robotics and intraoperative decisions are steadily increasing. While ML has benefited domains like DBS lead implantation, neuronavigation, pedicle screw placement, and tumor boundary classification, further innovation is on the horizon.

Diagnostic and imaging applications of ML in neurosurgery

ML can assist in the diagnosis and classification of major neurosurgical conditions. For epilepsy detection, CNNs have achieved performances of >96% accuracy using electroencephalography (EEG) data [75], extending to real-time seizure prediction [76]. Using CT angiography images for intracranial aneurysm detection, CNNs achieved an accuracy of 0.886 on independent internal validation. Aneurysms in the anterior communicating artery, anterior cerebral artery, vertebrobasilar artery, and middle cerebral artery were best predicted, with poor performance for tiny aneurysms (<3 mm) [77]. For the spine, ML has been used to diagnose fracture from CT [78] (accuracy 0.932), to detect foraminal stenosis using generative adversarial networks (GANs) for semantic segmentation [79] (mean average precision 0.837), and to diagnose osteoarthritis by DL from medical record data without any imaging [80] (accuracy 0.768). The use of ML for neurosurgical diagnostics is focused on tumor classification both pre and perioperatively. Such ML applications have predicted meningioma grade [81] (AUC 0.8895), non-invasive tissue classification by SVM from Hyperspectral Imaging [82], intraoperative tumor classification by DNA methylation profile [83] (accuracy 89%), and preoperative tumor classification by CNN from MRI-only data [84] (accuracy 0.9656).

Medical data including medical records, imaging (CT, MRI, EEG, hyperspectral imaging), and intraoperative tissue, serve as inputs to ML models for the diagnosis of neurosurgical conditions. However, ML and feature selection algorithms allow for radiomics: the discovery and development of clinically explainable radiologic features that are useful for prediction. For traumatic brain injury prediction and lesion classification, features including shape, intensity, and texture biomarkers were isolated and used independently, demonstrating the validity of these as radio markers for lesion classification [85]. For Gamma Knife radiosurgery, ML was used to discover features to predict surgical effectiveness, finding higher zone percentage as a radiomarker for achieving local tumor control in brain metastasis [86]. Feature-based ANNs were used to discover radio-markers for preoperative tumor differentiation between skull base chordoma and chondrosarcoma for preoperative planning, extracting the potential radiomarkers from preoperative MRI imaging, and by using only seven of these identified radiomarkers achieved an AUC of 0.93 [87].

State-of-the-art applications: GANs and federated learning

Generative Adversarial Networks

The applications of ML to diagnostics in neurosurgery extend beyond disease prediction, innovating the imaging methods themselves. Using GANs, MRI conversion to CT has been demonstrated in the brain [88-90] and spine [91]. A GAN generates images by training two DL models at the same time: one to generate synthetic images and another to discriminate between real and synthetic images, both competing with each other to simultaneously improve. Synthetic CT scans have demonstrated the ability to validate the downstream application of dosimetric determination for stereotactic brain radiotherapy [92], allowing for the potential conversion of MRI/CT workflow to MRI-only workflows for diagnostics and radiosurgical planning. GANs applied to positron emission tomography imaging improved spatial resolution [93] and dose reduction [94]. Lastly, GANs have allowed for the consideration of multiple imaging modalities, improving Alzheimer’s disease diagnosis by synthetically constructing missing imaging information and using a downstream CNN to combine the real and synthetic information to predict the diagnosis [95] (AUC > 0.87).

Federated Learning

A fundamental barrier in ML applications to neurosurgery is the lack of available "big data," mainly due to the cost of storage and the preservation of patient privacy. This preservation prohibits the sharing of medical data across institutions, restricting the ability to build large datasets necessary to train DL models and restricting algorithms to learn from data at one institution [96]. This “domain shift” in turn results in internally validated algorithms failing to generalize and externally validate [97]. One solution, synthetic learning, is to share synthetic data that represents the clinical information of patient data but preserves its privacy; efforts have begun to use GANs to generate synthetic data and to allow sharing across institutions. This approach has been proven to be unbiased and downstream models trained on the synthetic data have achieved high performance on brain tumors [98], nuclei segmentation [98], and spine radiograph abnormality classification [99]. The current most popular method to preserve data privacy while allowing for the training of complex networks across institutions is termed federated learning (FL). In FL, private data never leaves an institution. Instead, a basic ML model is trained on the institution's data, and statistics from the updated model are sent back to a central system. The aggregation of these results across many institutions may send an updated model that better represents patient populations, fine-tuned by the specific institutions before application. Such FL schemes in neurosurgery have demonstrated success, outperforming locally trained models, in intracerebral hemorrhage detection on CT scans [100] (AUC 0.9487) and automatic tumor boundary detection for glioblastoma [101]. The main restriction of FL is in rare data environments, where the initial single institution models cannot learn meaningful information. Future work must develop technical solutions to this problem as many applications of ML in neurosurgery have limited patient volume.

Large Language Models 

ChatGPT, a large language model (LLM) trained by OpenAI, has demonstrated the ability to pass neurosurgical boards. Similarly trained LLMs, such as NYUTRON, have been shown to achieve high performance for common neurosurgical outcome prediction tasks such as readmission prediction. At this cutting edge of ML in neurosurgery, motivated by the increasingly "black box" construction of these models and their training, it becomes essential to investigate training strategy, bias analysis, and hallucinations of the models (Table 5).

**Table 5:** Large language models in neurosurgery NYU: New York University; EHR: Electronic health records; GPT: Generative pre-trained transformer

Paper/model	Input variables	Training data source	Performance measurement	Hallucinations mentioned	Common hallucinations	Translated	Bias investigated
NYUTRON [102,103]	Text in clinical records	NYU EHR records	Mortality prediction, Comorbidity index, readmission prediction	No	No	No	Age, race
GPT-4 ChatGPT and Bard [104]	The 149-question self-assessment neurosurgery examination indications examination	Both supervised and unsupervised learning techniques on a large corpus of Internet text data	Score on neurosurgical examination	Yes	Incorrect image Analysis in test questions	No	N/A

## Conclusions

While ML is already impacting patient care, most of the developments discussed in this review have not been translated at a large scale. A primary issue is that these algorithms lack generalizability. While efforts toward standardization in AI research reporting are commendable and have led to significant improvements, the unique challenges in neurosurgery, such as differences in patient cohorts, pathology and outcome definitions, and imaging modality generation differences, highlight the need for tailored approaches to standardization in this field. Data sharing and technical solutions including FL should help transition to "big data" and with that, the increase of ML assistance in direct patient care in neurosurgery. As both algorithms and cases become more complex, incorporating inputs of imaging, video, and genomics, it is essential that neurosurgeons consider the technical elements of the proposed methods. As large language models and ChatGPT begin to be translated to neurosurgery, the translation of these models should be clinician-led and necessitate clinical metrics to carefully validate these tools while developing them to improve the quality of care.
